# NIMEFI: Gene Regulatory Network Inference using Multiple Ensemble Feature Importance Algorithms

**DOI:** 10.1371/journal.pone.0092709

**Published:** 2014-03-25

**Authors:** Joeri Ruyssinck, Vân Anh Huynh-Thu, Pierre Geurts, Tom Dhaene, Piet Demeester, Yvan Saeys

**Affiliations:** 1 Department of Information Technology, Ghent University – iMinds, Gent, Belgium; 2 Department of Electrical Engineering and Computer Science & GIGA-R, Systems and Modeling, University of Liège, Liège, Belgium; 3 School of Informatics, University of Edinburgh, Edinburgh, United Kingdom; 4 Laboratory of Immunoregulation, VIB Inflammation Research Center, Gent, Belgium; 5 Department of Respiratory Medicine, Ghent University, Gent, Belgium; Leibniz-Institute for Farm Animal Biology (FBN), Germany

## Abstract

One of the long-standing open challenges in computational systems biology is the topology inference of gene regulatory networks from high-throughput omics data. Recently, two community-wide efforts, DREAM4 and DREAM5, have been established to benchmark network inference techniques using gene expression measurements. In these challenges the overall top performer was the GENIE3 algorithm. This method decomposes the network inference task into separate regression problems for each gene in the network in which the expression values of a particular target gene are predicted using all other genes as possible predictors. Next, using tree-based ensemble methods, an importance measure for each predictor gene is calculated with respect to the target gene and a high feature importance is considered as putative evidence of a regulatory link existing between both genes. The contribution of this work is twofold. First, we generalize the regression decomposition strategy of GENIE3 to other feature importance methods. We compare the performance of support vector regression, the elastic net, random forest regression, symbolic regression and their ensemble variants in this setting to the original GENIE3 algorithm. To create the ensemble variants, we propose a subsampling approach which allows us to cast any feature selection algorithm that produces a feature ranking into an ensemble feature importance algorithm. We demonstrate that the ensemble setting is key to the network inference task, as only ensemble variants achieve top performance. As second contribution, we explore the effect of using rankwise averaged predictions of multiple ensemble algorithms as opposed to only one. We name this approach NIMEFI (Network Inference using Multiple Ensemble Feature Importance algorithms) and show that this approach outperforms all individual methods in general, although on a specific network a single method can perform better. An implementation of NIMEFI has been made publicly available.

## Introduction

Transcriptional regulation is a key mechanism for cells to accomplish changes in gene expression levels. As a consequence, deciphering the structure of the gene regulatory network (GRN) is crucial to gain insights in biological processes of interest or the pathology of a cell. The availability of large collections of gene expression compendia offer the potential to infer the network topology in high-throughput and on a large-scale. As a consequence, many computational tools to infer GRNs from expression data have been developed and are being used in practical use cases [1].

However, inferring the GRN from expression data is a severely underdetermined problem, as the amount of possible interactions largely exceeds the number of available measurements. Coping with this underdetermination has turned out to be a very difficult problem and has led to the development of an overwhelming amount of algorithms which use different strategies to overcome this inherent difficulty. Not only do these algorithms differ in the success they have to elucidate the network, they strike a balance between complexity and scalability and their predictions can be highly complementary [2], [3].

Algorithms that focus on inferring the topology of large GRNs typically calculate pair-wise measures between genes. Early methods used Pearson€s correlation coefficient [4], but failed to identify non-linear relationships between genes. To capture these more complex dependencies, information theoretic measures have been proposed. In particular many models infer networks using the mutual information between a pair of genes as a measure [5]. These methods generally suffer from predicting many false positive links due to indirect effects and consequently various refinements have been proposed. CLR [6] corrects the predictions based on the specific background distribution of all mutual information scores. The ARACNE algorithm [7] uses the Data Processing Inequality on every triplet of genes to filter out indirect effects. MRNET [8] builds on the maximum relevance, minimum redundancy concept [9] using an iterative feature selection scheme. Finally, C3NET [10] and its ensemble extension BC3NET [11] try to avoid inferring indirect effects by only predicting a link between two genes if the mutual information between genes is at least maximal for one of the genes with respect to all other.

More recently, the ANOVerence [12] algorithm proposed the 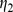 score as an alternative measure to evaluate dependencies between genes. The 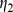 score is a non-parametric and non-linear correlation coefficient derived using ANOVA.

Finally, the TIGRESS [13] method solves the network inference problem by using a feature selection technique (LARS) combined with stability selection.

Various comparative studies have been performed which evaluate network inference algorithms [3], [14]–[16]. Due to the large variety of algorithms, these studies typically focus on a small subset of techniques and aim to derive interesting properties. Large scale evaluations of techniques have been performed in the context of the DREAM (Dialogue for Reverse Engineering Assessments and Methods) challenges [17]. DREAM aims to stimulate research in the field and provide researchers with benchmark datasets to validate their work. Community-wide network inference challenges where participants were invited to run their algorithms on blinded datasets have been organized. These challenges are the most comprehensive assessments of GRN inference algorithms.

In both the DREAM4 in silico 100 multifactorial challenge and the latest DREAM5 network inference challenge [18]–[20] the overall top performer was the GENIE3 algorithm [21]. This method approaches the network inference problem by decomposing it into a separate regression problem for each possible target gene. Next, using a tree-based ensemble method, an importance measure for each predictor is calculated and a high feature importance is used as an indication that a link is present between the predictor and the target gene in the GRN.

Motivated by the success of GENIE3 and other ensemble methods based on feature importance, such as TIGRESS, we wish to explore the potential of several other ensemble feature importance techniques in the regression decomposition setting. We present a general framework which casts any feature selection algorithm into an ensemble setting by taking random subsamples of varying size of both the experiments and the potential regulatory genes. Furthermore, given the known complementary of GRN inference methods [17], we explore if it is beneficial to combine the predictions of several ensemble feature importance scoring algorithms as opposed to using a single one.

Summarizing, we name this approach NIMEFI (Network Inference using Multiple Ensembles of Feature Importance algorithms) and compare the performance of this new method to several recently proposed state-of-the-art techniques.

## Materials and Methods

### Problem Statement, Evaluation and Data Sources

In this paper we focus on the inference of the directed topology of large gene regulatory networks using gene expression data. Self-regulating interactions are not taken into consideration. As input data, we assume a compendium containing several gene expression measurements obtained from one or more experiments. We make no further assumptions about whether the data was compiled using gene-knockouts, multifactorial pertubations, steady-state measurements or any other experimental settings, nor do we take any time-related information into account. Although the directionality of a regulatory link is hard to infer without extracting specific information in interventional or time-series data, we opt for a directed topology setting throughout this paper as this was the setting of the DREAM challenges and it allows for a fair comparison to other algorithms. Furthermore, it has been shown that the GENIE3 algorithm is able to predict directionality using the same data handling strategy [21]. Lastly, we allow for an optional limitative list of known regulatory genes as input data, in which case no outgoing links from other genes are allowed as predictions.

As such, let us define a learning sample (LS) from which to infer the GRN as a matrix of 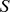 rows by 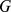 columns, in which each row can be interpreted as the expression values of all 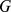 genes in one of the 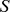 available samples
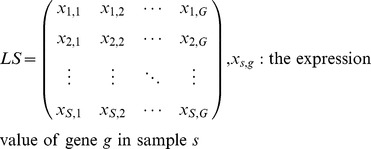



The desired output of the algorithm is then defined as a (fully connected) directed graph in which each node corresponds to a single gene and an edge between a certain node 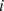 and another node 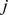 indicates that gene 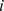 regulates the expression of gene 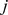. For each possible edge in the network a score is given which represents the confidence that this is a true regulating link.

A recurrent problem among GRN inference methods is selecting a suitable cut-off value to transform the obtained ranking into an actual network structure. Some methods (e.g. [10], [11]) try to automatically select a network instead of a ranking while others leave the decision to the end-user. In this work, we do not automatically select a threshold value and instead will focus on the ranking. We believe that it is beneficial to the end-user to explore the network at various threshold levels. Some parts of the network can have a weaker signal in the gene expression set, but still be relevant to the user. As we focus on evaluating the ranking as opposed to a network, we will adopt the widely used DREAM5 procedure to score network predictions. In this procedure, all edges are sorted by decreasing confidence score and only the top 100,000 predictions are retained. Next, only considering the ranks, both the Area Under the Receiver Operating Characteristic curve (AUROC) and Area Under the Precision-Recall curve (AUPR) are calculated with respect to a known gold standard.

We will evaluate all algorithms using five gene expression sets provided by the DREAM4 in silico 100 multifactorial challenge (DREAM4) and two gene expression sets from the DREAM5 network inference challenge (DREAM5). Furthermore, we created several artificial gene expression datasets using both the SynTReN [22] (SYNTREN-100) and GeneNetWeaver (GNW-100,GNW-200) [18], [23] simulators. Table 1 provides an overview of the various datasets.

**Table 1:** Characteristics of the different datasets used for evaluation.

Name	# Samples	# Genes	# Regulatory genes	Type
DREAM4 [5 net. - 1 comp. each]	100	100	100	Artificial
DREAM5 artificial [1 net. - 1 comp.]	805	1643	195	Artificial
DREAM5 *E. coli* [1 net. - 1 comp.]	805	4511	334	Real
SynTRreN-100 [1 net. - 20 comp.]	100	100	100	Artificial
GNW-100 [1 net. - 20 comp.]	100	100	100	Artificial
GNW-200 [15 net. - 1 comp. each]	200	200	200	Artificial

‘net.’ indicates the amount of different underlying network topologies in the dataset, while ‘comp.’ indicates the amount of expression compendia associated with these network topologies.

The DREAM4 dataset consists of five artificial networks, each of size 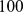 (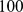 genes described by 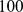 experiments). These datasets were created for the DREAM4 in silico 100 multifactorial challenge and aim to mimic samples from multifactorial perturbation data, which is defined as static steady-state expression profiles achieved by slightly perturbing all gene expression values at the same time. In addition, two more datasets were used from the DREAM5 network inference challenge. The first is an artificial dataset consisting of 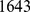 genes, including a known list of 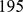 potential regulatory genes. No genes, other than these included in the list are regulatory genes in the gold standard used for validation. The topology of the in-silico network is based on known GRNs of model organisms. The compendium consists of various experimental settings. The second DREAM5 dataset describes a large real expression compendium of *E. coli*. This dataset contains a known list of 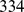 potential regulatory genes, and consists of measurements of 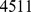 genes obtained in various experimental conditions.

To further test our findings, we created two network topologies of 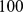 nodes using an underlying *E. coli* network, one using SynTReN and one using GNW. For both underlying networks, 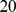 artificial expression compendia of 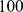 samples each were created using default settings. Lastly, using GNW and the same settings, we created fifteen different network topologies consisting of 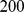 nodes, for each network a dataset of 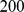 samples was simulated, again using default settings.

### Ensemble Feature Selection Techniques

Feature selection is an important preprocessing step in many machine learning applications, where it is often used to find the smallest subset of features that maximally increases the performance of the model. Other benefits of applying feature selection include the ability to build simpler and faster models using only a subset of all features, as well as gaining a better understanding of the processes described by the data, by focusing on a selected subset of features [24], [25]. Three types of feature selection techniques can be distinguished. Filter methods operate directly on the dataset, and provide a feature weighting, ranking or subset as output. These methods have the advantage of being fast and independent of the model, but at the cost of inferior results. Wrapper methods perform a search in the space of feature subsets, guided by the outcome of the model. They often report better results than filter methods, but at the price of an increased computational cost. Finally, embedded methods use internal information of the model to perform feature selection (e.g. use of the weight vector in linear models). They often provide a good trade-off between performance and computational cost. Recently, the concept of ensemble feature selection (EFS) was introduced in various problems. Just like ensemble models for classification and regression, EFS performs feature selection by combining different feature selection algorithms, usually obtained by bootstrapping, and then aggregates their results as the final output. EFS often results in better performance and more stable feature rankings than a single feature selection technique [26]–[28].

### Generalizing GENIE3

**Figure 1:**
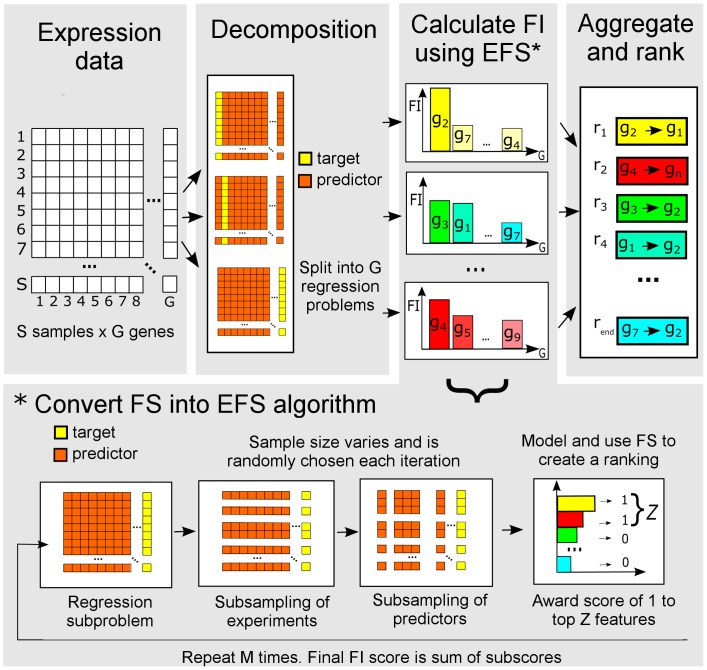
Overview of the EFS approach to the network inference task. The problem is split into independent regression subproblems for each gene in the network. Next feature importance (FI) scores are calculated in each subproblem for all possible regulatory genes with respect to the target gene using an ensemble feature selection (EFS) method. These FI scores are then assigned as the weight of an edge in the network from the regulatory gene to the target gene. Finally, all weights are aggregated across the subproblems, creating a global confidence ranking of edges. We cast any feature selection (FS) method which can provide a ranking into an EFS method by taking random samples of varying size of both the experiments and the possible predictor genes and assigning a score of 1 to the top features in the ranking.

GENIE3 decomposes the network inference problem between 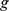 genes as 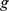 separate regression problems. Each regression problem aims to predict the expression values of a particular target gene using the other genes as input genes. Using feature selection in this regression context then amounts to finding the other genes that are most indicative in modeling the expression values of the target gene, thus providing evidence of important regulators of the target gene. GENIE3 provides a ranking of the regulators of the target gene by deriving a weight for each regulator based on an ensemble of tree-based regression models, such as random forests [29]. Conceptually however, any kind of feature selection technique could be used to provide this ranking. The general principle of such a feature selection based approach to network inference is depicted in Figure 1 and can be summarized as follows:

1. For 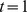 to 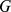

  - Build a regression model predicting the vector of expression values of the target gene 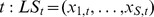 using the learning sample matrix without the target gene values: 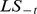. Each column representing a possible predictor.
  - Use a feature selection technique FS to compute a feature importance (FI) value for each predictor column (gene) 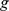 in 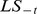 : 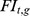.
2. Aggregate the 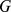 individual regression problems to get a global ranking of all possible regulatory links in the network.

The feature selection technique used here is thus supposed to be able to compute a feature importance value for each input gene (feature) of the regression problem associated to gene 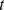. In principle, any feature selection technique that is able to deal with a continuous output and returns some kind of feature importance measure or score can be used in this framework.

Instead of using a single feature selection algorithm to compute the importance values we could also use an ensemble of 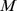 feature selection algorithms 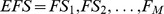 to calculate the importance values. The GENIE3 approach in the random forest setting is just a special case of such a more general ensemble setting, combining the bagging procedure to generate different regression tree models with random sampling of the variables within each regression tree node.

In general, any appropriate feature selection technique in this regression context can be easily cast into the EFS setting by generating 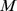 subsamples of the original learning sample LS, e.g. using regular subsampling or subsampling with replacement (bootstrap sampling). More specifically, we used the following approach to transform any FS algorithm which can produce a ranking to create an EFS method that produces FI scores for a regression subproblem with target gene 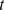 (Figure 1) :

1. For 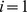 to 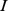

  - Take a subsample without replacement of size 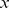 of the samples (rows) of 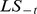 and the target vector 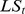; where 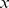 is a uniformly randomly generated integer number between 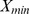 and 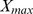.
  - Further reduce the matrix and target vector in size by taking a subsample of size 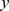 without replacement of the possible genes (columns); where 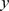 is a uniformly randomly generated integer number between 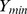 and 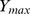.
  - Rank the features in the reduced predictor matrix by their ability to predict the reduced target vector using a FS technique of choice.
  - Assign a score of ‘
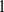
’ to the top 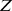 features of the ranking and a score of ‘
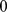
’ to all other.
2. Sum the scores across all iterations and use these sums as the final FI scores for each predictor in the regression subproblem.

In the above algorithm the parameters 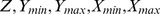 are typically set to default values of 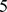, 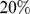, 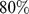 of the amount of predictors and 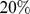, 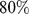 of the amount of experiments respectively. 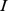 is set to allow convergence. We explore and discuss the effect of these parameters on the algorithm performance in the results section. However, the general idea is to create subsamples of varying sizes, effectively searching for both global connections between genes as well as effects which can only be seen in a small amount of experiments. By also sampling the predictors in each iteration, we avoid the problem of dominant genes masking possible secondary interactions. For computational reasons we imposed a maximum value of 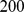 on the upper bounds.

### Calculating Feature Importance Values Using Different Machine Learning Techniques

In our experiments, the following machine learning algorithms in the regression context were used to calculate feature importance values. Two linear methods: linear support vector regression (SVR) and regularized regression using the elastic net (EL) as well as their respective ensemble versions (E-SVR, E-EL). We also used symbolic regression (SR), an inherently ensemble, non-linear method. For each of these methods we briefly describe how the feature rankings were calculated. For comparison reasons we also added an ensemble random forest regression (E-RFR) variant, differing from GENIE3 only due to the use of the subsampling scheme described earlier instead of a single regression for each target gene. In all of our experiments we used GENIE3 in random forest mode with the number of trees (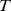) set to 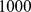 and the number of randomly selected genes at each node of the tree (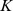) set to 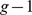.

Support vector regression: from the Lagrange multipliers 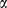, the support vectors 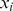 and the training labels 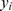 one can calculate the weight vector 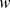 in case of a linear kernel as:
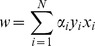



The absolute value of the weight 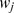 of the weight vector 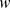 corresponding to a certain feature 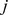 can be used to assess feature importance, as it is clear that small values will have less impact on the predicted value [24]. We rank features in a single step as opposed to using the recursive feature elimination scheme of [31] for computational reasons. All experiments were conducted using the libsvm [30] package for R, using epsilon regression and a linear kernel function. The cost of constraint violation parameter C was set to the default value of 1, all other parameters were also set to their respective default values.

Elastic net: the absolute values of the coefficients of the predictors after a fit with a regularized linear model using the elastic net penalty function [32] are used as a measure for feature importance. The elastic net offers a compromise between ridge regression and the LASSO. The mixing coefficient, 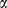 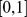, determines the tradeoff between the L1-norm and L2-norm regularization. If 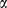 is set to 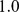, it corresponds to the LASSO penalty, 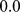 corresponds to the ridge penalty. The 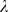 parameter is defined as the regularization parameter and was determined by cross-validation. Experiments were conducted using the glmnet package for R [33] with 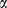 set to 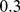. We explored several other settings for the 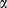 parameter. Setting 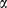 to 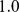 had a negative effect on the performance, because less than 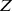 features received a non-zero score in the regression subproblem. We did not notice any major performance differences between other settings of 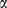.

Symbolic regression: this method aims to capture the input-output relation with an algebraic expression found through a genetic programming approach. In each iteration of the algorithm a large set of formulae, the population, is explored and regenerated using modifications such as crossover and mutation operations. We used pareto-aware symbolic regression [34] which utilizes multiple objectives to assess models. In our case, a Pareto-front is used which strikes a balance between the complexity of a formula and the goodness of fit. Next we determine a feature importance score for each score by a presence-based measure [35]. As irrelevant variables will cause extra complexity without lowering the error measure of the mode, these variables are discouraged to be included in the population. As such, the final population after a modeling run will likely only contain the most relevant variables. We use the percentage of the population in which a feature is present as the feature importance score. Other feature scoring approaches were also explored, but achieved similar or worse results. Experiments were conducted using the proprietary DataModeler software (http://www.evolved-analytics.com/) using four independent evolutions.

### TIGRESS

We included the results of TIGRESS in all our performance comparisons. The performance scores of TIGRESS for the DREAM5 dataset were taken from the DREAM5 challenge results. All other performance metrics were obtained by running TIGRESS using the GenePattern platform (dream.broadinstitute.org) with default parameter settings and 1000 iterations.

### NIMEFI

To explore the known complementarity of GRN inference methods, we create new predictions by combining the predictions of several algorithms. In particular, we focus on the combination of several FI scoring algorithms as opposed to only using a single algorithm, an approach which we named NIMEFI. In order to create combined predictions, we aggregate using Borda count, which in this setting is equal to averaging rankwise across predictions to obtain a newly predicted rank for each possible regulatory interaction. We do not impose a prior cut-off value to the individual prediction rankings of the methods before merging. Other aggregation strategies such as minimum rank or median rank were explored but were omitted from this paper as the results were either similar or worse than averaging rankwise.

## Results

### Performance Comparison on the DREAM4 Size 100 in Silico Multifactorial Dataset

Table 2 lists the performance of several algorithms on the DREAM4 multifactorial dataset. The p-values listed in the table represent the probability that a given or larger area under the curve value is obtained by random ordering of the links in the ranking. For comparison reasons, we include the scores of the TIGRESS algorithm. A first observation is that the ensemble setting seems to be key to the network inference problem. Both the single-step versions of the ELand SVR are unable to compete with the other algorithms. However, when casting these algorithms into the ensemble setting, their performance increases drastically reaching similar scores than those of GENIE3 and TIGRESS. The performance of Symbolic Regression is also similar to all the EFS-methods. Both Symbolic Regression and GENIE3 can be classified as inherently ensemble feature importance scoring algorithms. A second observation which can be made is that GENIE3 outperforms E-RFR, although in both algorithms random forest regression is used. GENIE3 calculates the feature importance based on the variance reduction due to splitting on a feature [36]. Our results indicate this measure outperforms the counting measure implemented in the subsampling scheme. We believe this is partially because the variance reduction measure allows for a more objective comparison between different regression problems, allowing for a better aggregation towards a global ranking across the different regression subproblems. A final observation which can be made is the seemingly overall better performance of the averaged predictions of multiple ensemble feature importance algorithms as opposed to predictions of a single one. All combined predictions show an increase in AUROC score, while retaining a similar score for AUPR. In the last column the standard deviation of the performance scores over five runs is shown. The standard deviation is close to 0 for all algorithms. Both EL and SVR are deterministic, the other algorithms introduce randomness but output stable results due to their ensemble nature.

**Table 2:** Performance comparison of several algorithms on the DREAM4 in silico multifactorial dataset.

Method	Metric	Net1	Net2	Net3	Net4	Net5	Avg.
EL	AUROC	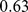	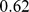	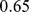	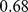	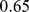	**0.64** ( 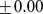 )
	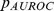	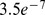	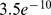	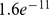	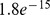	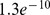	
	AUPR	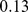	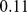	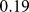	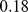	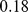	**0.15** ( 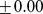 )
	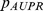	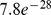	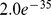	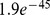	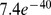	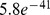	
E-EL	AUROC	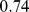	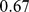	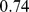	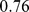	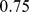	**0.73** ( 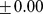 )
	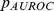	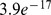	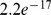	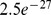	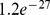	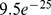	
	AUPR	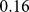	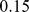	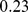	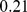	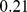	**0.19** ( 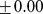 )
	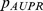	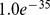	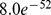	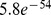	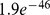	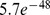	
SVR	AUROC	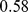	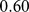	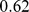	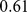	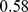	**0.57** ( 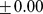 )
	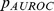	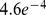	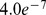	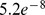	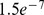	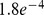	
	AUPR	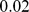	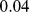	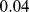	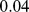	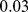	**0.03** ( 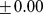 )
	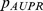	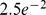	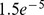	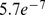	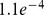	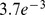	
E-SVR	AUROC	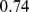	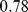	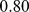	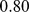	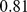	**0.78** ( 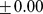 )
	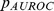	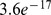	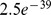	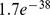	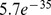	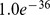	
	AUPR	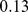	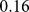	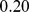	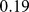	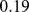	**0.17** ( 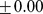 )
	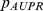	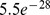	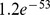	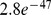	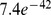	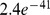	
SR	AUROC	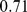	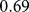	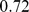	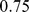	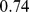	**0.72** ( 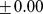 )
	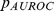	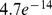	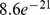	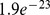	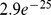	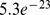	
	AUPR	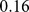	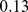	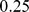	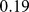	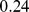	**0.19** ( 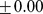 )
	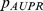	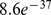	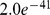	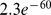	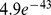	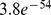	
E-RFR	AUROC	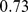	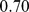	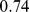	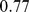	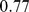	**0.74** ( 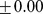 )
	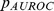	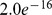	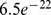	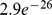	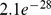	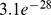	
	AUPR	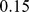	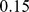	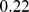	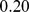	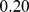	**0.18** ( 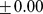 )
	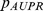	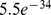	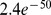	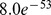	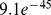	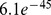	
GENIE3	AUROC	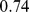	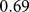	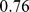	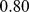	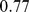	**0.75** ( 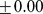 )
	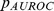	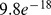	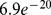	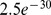	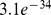	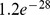	
	AUPR	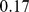	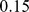	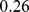	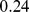	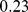	**0.20** ( 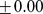 )
	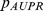	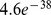	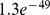	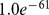	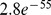	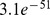	
TIGRESS	AUROC	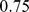	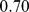	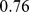	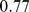	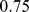	**0.76** ( 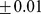 )
	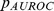	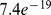	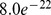	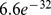	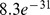	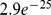	
	AUPR	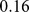	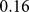	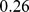	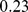	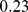	**0.20** ( 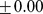 )
	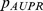	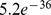	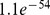	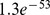	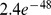	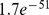	
GENIE3+E-SVR	AUROC	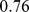	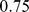	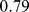	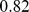	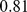	**0.79** ( 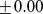 )
	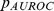	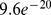	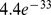	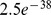	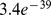	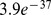	
	AUPR	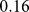	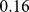	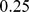	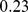	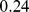	**0.20** ( 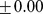 )
	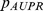	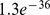	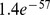	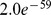	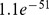	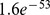	
ALL	AUROC	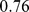	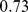	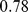	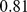	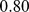	**0.78** ( 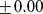 )
	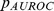	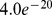	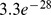	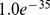	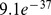	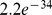	
	AUPR	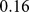	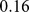	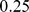	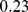	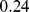	**0.20** ( 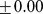 )
	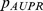	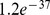	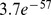	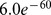	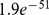	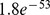	

(EL = Elastic Net, E-EL = Ensemble Elastic Net, SVR = Support Vector Regression, E-SVR = Ensemble Support Vector Regression, SR = Symbolic Regression, E-RFR = Ensemble Random Forest Regression). ‘+’ indicates rankwise averaging of several methods. ALL = GENIE3+E-SVR+E-EL. Standard deviation is shown between brackets. Ensemble variants, indicated with ‘E-’, were created using the subsampling scheme with default settings. We refer to the manuscript for an interpretation of the listed p-values.

### Performance Evaluation on the DREAM5 Dataset

Table 3 shows the performance on the DREAM5 datasets. The meaning of the p-values in this table is different from Table 2. They should be interpreted as the probability that a given or larger area under the curve value is obtained by a random prediction generated by creating a list of edges out of all the submitted networks in the DREAM5 challenge in the following way: for each row in the list, randomly choose an edge among all the edges in the submissions at that row. The final p-value was computed by extrapolation of a curve fitted to the normalized histogram of the area under the curve for an ensemble of random lists.

**Table 3:** Performance comparison of several algorithms on the DREAM5 dataset.

Method	Artificial				*E. coli*			
	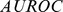	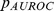	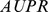	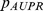	AUROC	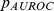	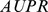	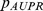
E-SVM	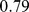	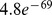	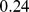	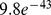	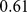	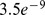	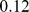	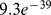
E-EL	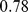	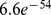	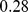	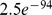	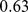	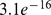	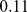	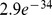
GENIE3	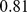	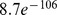	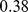	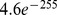	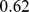	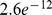	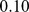	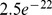
G+E-SVR	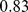	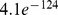	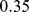	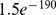	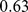	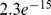	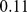	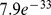
G+E-SVR+E-EL	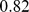	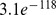	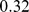	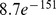	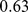	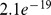	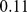	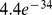
TIGRESS	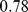	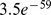	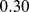	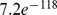	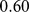	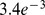	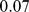	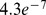
ANOVerence	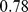	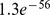	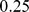	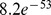	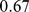	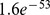	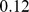	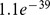

(E-EL = Ensemble Elastic Net, E-SVR = Ensemble Support Vector Regression, ‘+’ indicates rankwise averaging of several methods). We refer to the manuscript for an interpretation of the listed p-values.

We included the results of ANOverence, ranked second best overall in the DREAM5 challenge, with the best performance on the *E. coli* network. However, as opposed to GENIE3 and TIGRESS, ANOVerence does include meta-information of the microarray chips to guide the network inference process.

GENIE3 performs well on the artificial dataset, outscoring all individual methods. Note that in the original DREAM5 challenge results, GENIE3 reached a lower overall score because a different parameter setting was used for the number of input variables that are randomly chose at each node. The ensemble versions of EL and SVR are able to perform slightly better than ANOverence but are outperformed by TIGRESS with regard to the AUPR score.

The combinations GENIE3+E-SVR and GENIE3+E-SVR+E-EL are very competitive, reaching a higher AUROC score than GENIE3 but perform worse with regard to the AUPR score. Both methods however clearly outperform TIGRESS and ANOverence on this particular dataset.

On the biological *E. coli* dataset the individual methods, E-SVM, E-EL and GENIE3, show a similar performance. The combination predictions have a slightly higher score overall, but the difference is minimal compared to the individual methods. ANOVerence reaches the overall best score, making effective use of the available meta-information of the microarray chips.

### Performance Evaluation on the SyNTreN and GeneNetWeaver Datasets

**Figure 2:**
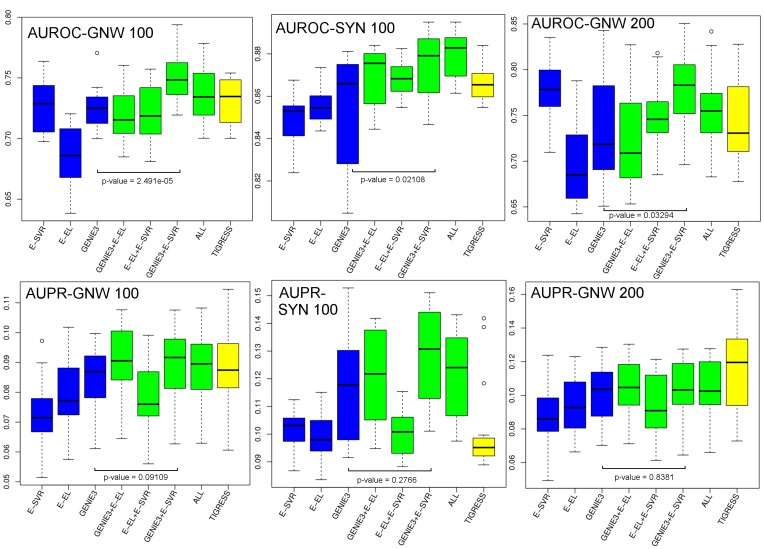
Boxplots of AUROC and AUPR scores on the three artificially created datasets. Shown in blue is the performance of three individual algorithms: GENIE3 and the ensemble versions of support vector regression and the elastic net (E-SVR, E-EL). Indicated in green the results after rankwise merging the individual methods and in yellow the performance of TIGRESS.Indicated in the figure are the results of Mann-Withney U-tests between GENIE and GENIE3+E-SVR (sample size 20 for GNW-100 and SYN 100, sample size 15 for GNW-200) showing that the AUROC scores are significantly improved.

The previous experiments were performed on benchmark data and the sample size was insufficient to determine if the NIMEFI approach outperformed the individual ensemble methods. In order to further explore if there is a significant difference in performance, we performed additional experiments on synthetic generated data of which the results can be seen in Figure 2. The boxplots show the AUROC and AUPR scores on three artificial datasets as described in the previous section. For these datasets we show the results of E-EL, E-SVR and GENIE3 and all possible combinations of these three methods. Again for comparison reasons, the results of TIGRESS are shown. We omitted the Symbolic Regression algorithm from the comparison as the computational complexity of this algorithm greatly exceeds all others, resulting in quickly deteriorating performance as the expression matrix size increases. The results for E-RFR were also omitted from the figure in favor of GENIE3 due to a consistent better performance of the latter method.

Again, as on the DREAM4 dataset, the ensemble versions of EL and SVR seem the be very competitive with GENIE3 without an overall winner between these three algorithms surfacing over all datasets. Interestingly, is the performance increase by rankwise combining predictions as seen in GENIE3+E-SVR and GENIE3+E-SVR+E-EL (ALL). Especially the combined prediction of GENIE3+E-SVR seems most promising. Indeed comparing GENIE3 to GENIE3+E-SVR we notice a significant improvement in AUROC score in all three datasets (Mann-Withney U-test, GENIE and GENIE3+E-SVR, sample size 20 for GNW-100 and SYN 100, sample size 15 for GNW-200, p-values: 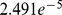, 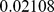, 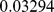), while the AUPR values are not significantly different (Mann-Withney U-test, same settings, p-values: 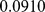, 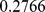, 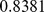).

Combined with the results of the previous section, this suggests that overall it is beneficial to use multiple ensembles feature selection techniques, as they provide better and more consistent performance among different datasets.

### Influence of the Parameter Settings of the Subsampling Scheme

In the previous results, the parameters 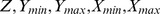 were always set to their respective default values of 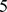, 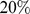, 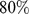, 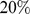 and 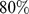. The parameter 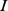 was set to 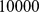 to allow for complete convergence. In this section, we explore various different settings for these parameters. We show average AUROC scores using the E-SVR variant on the DREAM4 in silico multifactorial dataset. The AUPR scores show similar behavior.

Table 4 shows the effect of only varying the 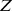 parameter, which controls how many predictors receive a score of ‘1’ in a single feature ranking of a subsample regression problem. The results indicate that for low values of 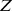, the performance is stable but consistently degrades towards higher values for 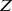. This result can intuitively be explained by the fact that for higher values of 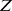 the algorithm loses discriminative power between the top ranked genes as all genes in the top 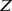 of the feature ranking will receive the same score of 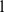. One could also try to determine 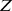 in an automated way using the feature importance scores in a single subsample regression problem. We explored this option by using an algorithm based on a Grubbs€ test for outliers to determine the optimal amount of predictors, however this lead to a significant decrease in performance across all datasets. We believe determining a cut-off value is a non-trivial problem in this context, as the distribution of the feature importance scores varies significantly across the regression subproblems. This is further complicated by the fact that some feature importance scores, e.g. the absolute value of the SVM feature weight vector, are hard to interpret outside of an ordinal context. Concluding, we advise a value of 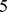 for the 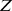 parameter for all datasets, as it is in the stable region. Furthermore, from a biological perspective, it is unrealistic to e.g set 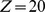 as this would imply that a gene is directly regulated by at least 20 other genes present in the subsample of predictors.

**Table 4:** Influence of the subsampling scheme parameter Z on the E-SVR AUROC score using the DREAM4 dataset.

Z rank threshold				
2	5	10	25	50
0.00	**0.79**	−0.02	−0.04	−0.08

Default setting indicated in boldface. Difference in AUROC score compared to the default setting is shown.

Next, we investigate the effect of the parameters 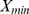 and 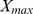, which control the size of the subsample of the experiments in the algorithm. Table 5 lists the performance for varying values. We notice that the performance suffers if only small subsamples (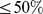) are included throughout the iterations. This is the case if 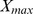 is set too low, especially in combination with a low 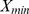. If the 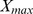 parameter is set to 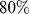 or 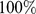 we notice a stable region in which the 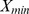 parameter has little effect on the performance. If both parameters are set to 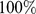, resulting in only subsampling the predictors in each iteration, a small performance drop can be seen. From a computational perspective, smaller subsamples are preferred as the regression subproblem is faster to solve. Taking both observations into account, we suggest a default value of 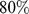 for 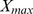 and a default value of 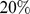 for 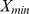.

**Table 5:** Influence of the subsampling scheme parameters 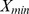 and 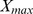 on the E-SVR AUROC score using the DREAM4 dataset.

	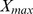				
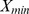	5	20	50	80	100
5	−0.12	−0.12	−0.07	−0.01	−0.01
20	/	−0.11	−0.05	**0.79**	−0.01
50	/	/	−0.01	0.00	−0.01
80	/	/	/	−0.01	−0.01
100	/	/	/	/	−0.02

Default setting indicated in boldface. Difference in AUROC score compared to the default setting is shown.

Furthermore, the effect of the parameters 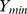 and 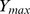 on the performance is shown in Table 6. These parameters control the size of the subsample of the possible predictors in each regression subproblem. The results indicate that the performance decreases with higher values for 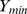 (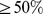), especially in combination with higher 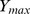 values. We believe the algorithms benefits from smaller predictor subsamples as it allows for alternative features to be picked up as important predictors in the regression subproblem, which would otherwise be missed because another (dominant) gene was used in the prediction model. Again from a computational perspective, smaller values for 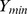 and 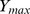 are preferred as it decreases the complexity of the regression subproblem. As such, we also advice for default values of 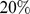 for 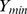 and a default value of 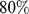 for 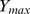.

**Table 6:** Influence of the subsampling scheme parameters 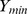 and 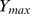 on the E-SVR AUROC score using the DREAM4 dataset.

	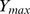			
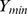	20	50	80	100
5	−0.01	0.00	0.00	−0.02
20	0.00	0.00	**0.79**	−0.02
50	/	0.00	−0.02	−0.05
80	/	/	−0.07	−0.10
100	/	/	/	−0.13

Default setting indicated in boldface. Difference in AUROC score compared to the default setting is shown.

**Figure 3:**
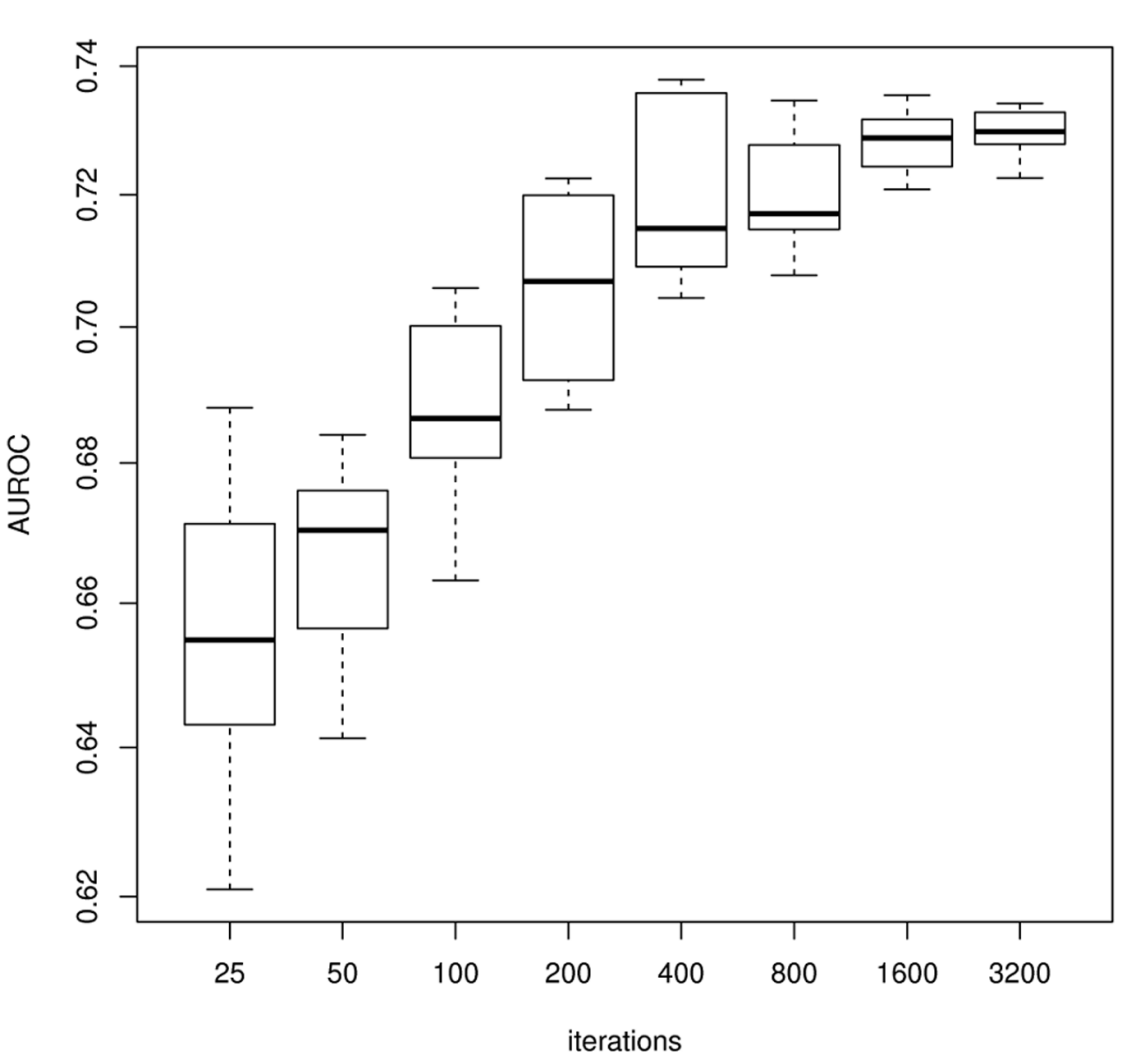
Boxplots of AUROC scores over ten runs with respect to the amount of iterations. The boxplots show the AUROC score over ten runs of the E-SVR algorithm on the first network of the DREAM4 dataset. The variance decreases as the amount of subsamples is increased, reaching a stable result at about 1500 iterations.

Lastly, we explore the effect of the number of iterations on the stability of the algorithm. Figure 3 shows eight boxplots over ten runs for different amount of total iterations. As desired, the performance variance between runs decreases as the amount of subsamples is increased, reaching an almost completely stable result at values above 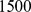 iterations.

### Additional Comparisons between Methods

**Figure 4:**
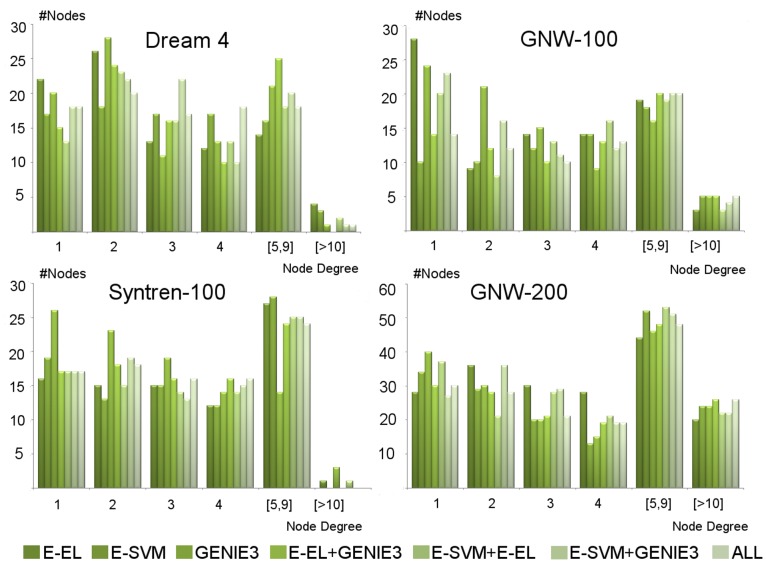
Node degree distribution of four network predictions selected across the different datasets. Networks predictions were interpreted in an undirected setting. The networks were created from the rankings by imposing a cut-off value close to the amount of true links in the corresponding gold network. Although the figure indicates that the node degree distribution can vary for the different algorithm predictions, there is no consistent pattern across the expression sets.

**Figure 5:**
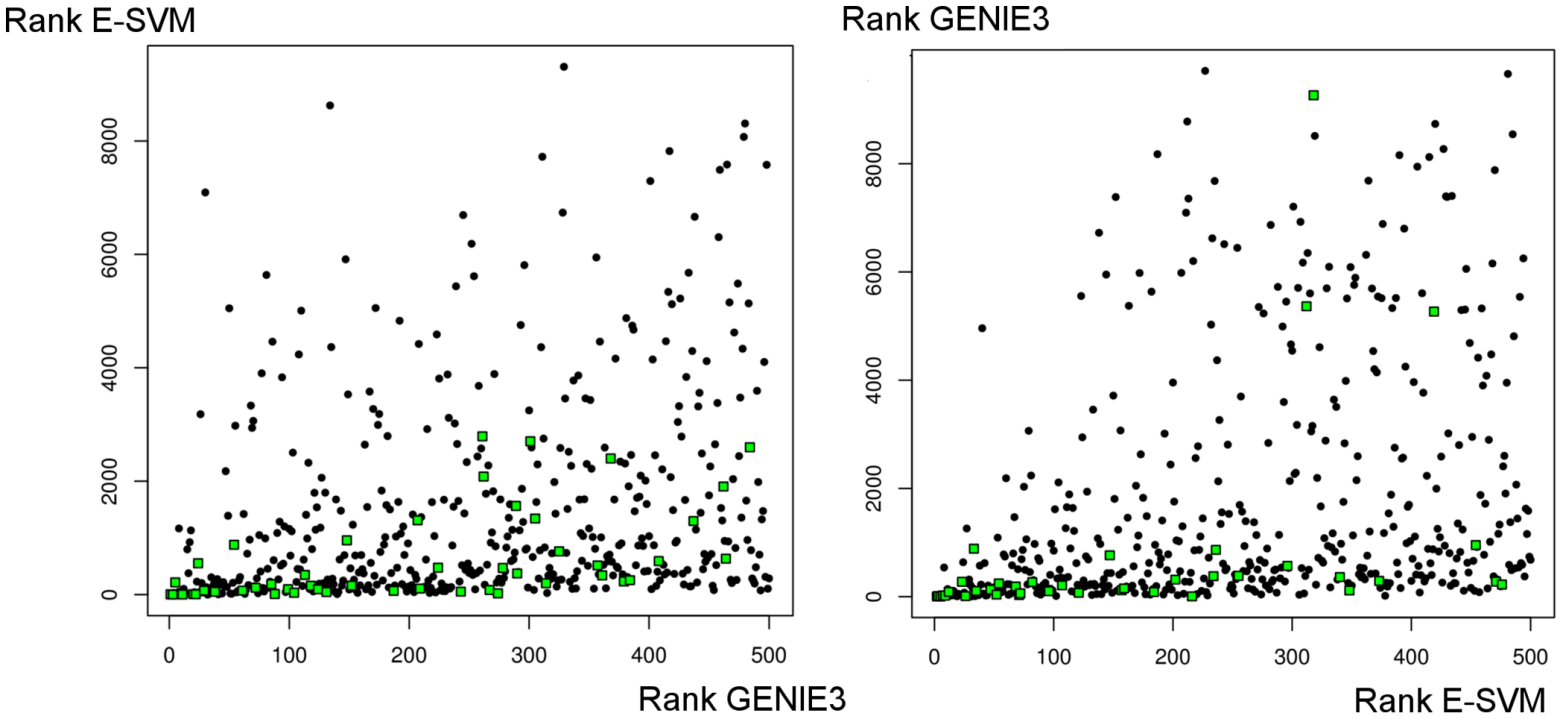
Comparison of the given rank at the edge level of two algorithm predictions. In the figure on the left we plot the rank of the top 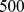 most confident links of the GENIE3 prediction versus the rank which these edges received in the E-SVM prediction. True positive links are indicated as green squares. Although the AUROC and AUPR scores of both methods are almost identical for this network, several top predicted edges by GENIE3, including true positives, appear much further down the ranking of E-SVM and vice versa.

We explored if there are other differences in the predictions of the investigated methods besides the performance. First, we analyzed if there were any biases in the node degree distribution of the inferred networks. We transformed the network predictions into an undirected setting by retaining the first edge in the ranking between each possible pair of nodes and removing the other. Next, networks were created from the undirected rankings by imposing several cut-off values. Figure 4 shows the node degree distribution of all algorithms on four networks selected from the different datasets. Here a cut-off value close to the amount of links in the underlying gold standard network was chosen (450 for GNW-200, 150 for all others). The figure indicates that the different methods can have a dissimilar node degree distribution on a certain network, however we found no clear bias associated with an algorithm across all network predictions. We notice that although the performance of two algorithms can be similar, the individual predictions seem to vary. Figure 5 illustrates this behavior further. In this figure we selected two predictions (GENIE3 and E-SVR) which had a similar performance score on a network in the GNW-100 dataset. Both algorithms had an AUROC score of 0.70 and an AUPR score of 0.08. We plotted the 500 top edges of the GENIE3 ranking versus the position they received in the E-SVR ranking and vice versa. True positive links are shown in green. For both plots, several links appearing at the top of the ranking for one prediction, are ranked at the very bottom of the other. This observation and the varying node degree distribution seem to hint that the predictions of the algorithms can show a different behavior, however it is non-trivial to quantify this difference.

**Figure 6:**
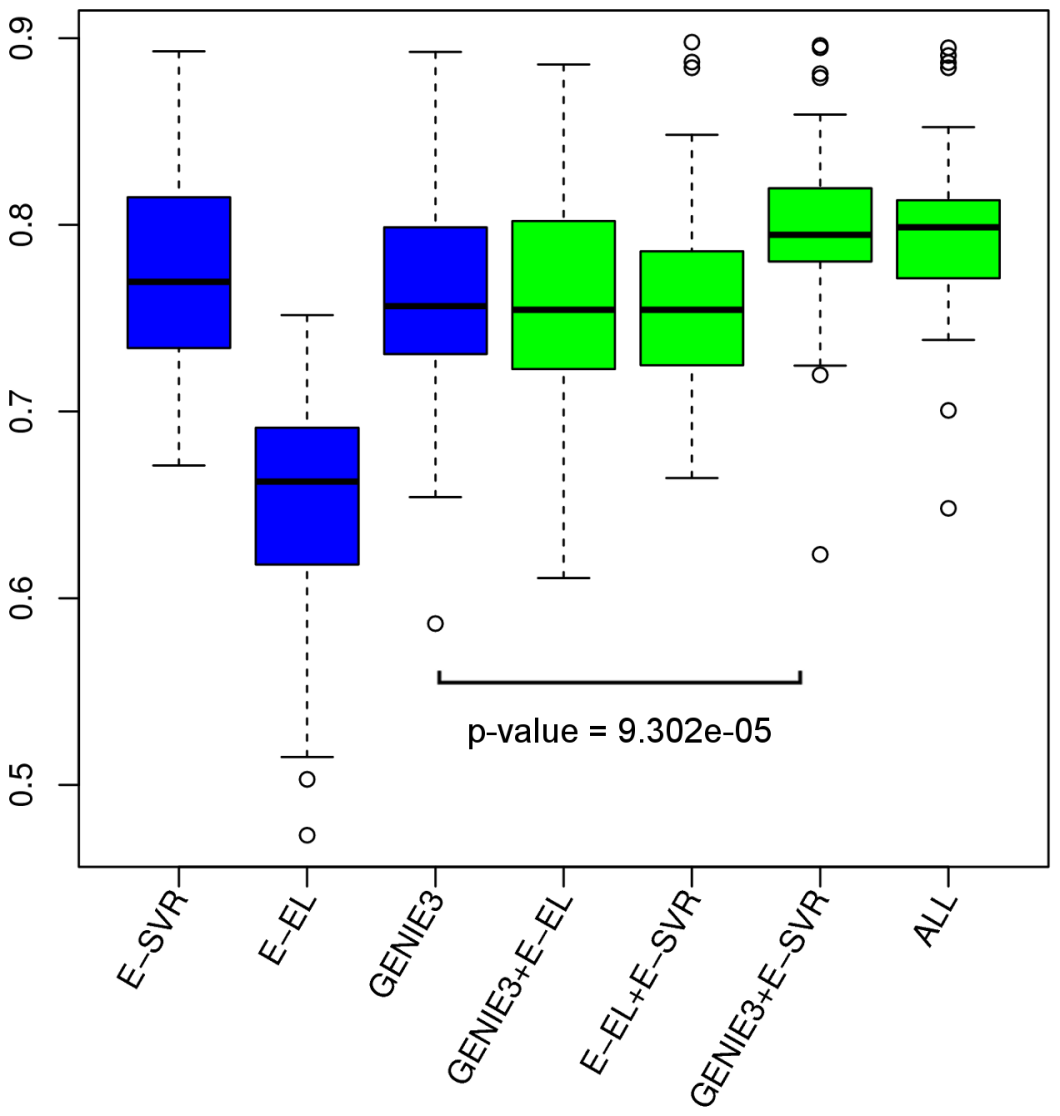
Boxplots showing the ability to predict the correct directionality of a true positive link. For all predictions we counted the amount of times a gold standard link 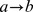 was ranked before the opposite link 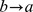, proportional to the total amount of links in the gold network. We performed this analysis for all networks in the DREAM4, GNW-100, SYNTREN-100 and GNW-200 datasets. The boxplots show the results for all algorithms. The GENIE3+E-SVR is significantly better at predicting the correct direction compared to GENIE3 (Wilcoxon rank sum test with continuity correction: GENIE3 and GENIE3+E-SVR, sample size 60, p-value = 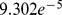).

Finally, we investigated the ability of the algorithms to predict the correct directionality of the link as in [21]. First, we removed all bi-directional links from the gold standard networks. Next, we counted the amount of times a gold link 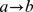 was ranked before the opposite link 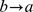, proportional to the total amount of gold standard links. We performed this analysis for all networks in the DREAM4, GNW-100, SYNTREN-100 and GNW-200 datasets. We omitted the two networks of the DREAM5 dataset as a list of regulatory genes was available. Figure 6 shows a boxplot of the directionality scores for all algorithms on the remaining 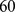 networks. A first observation is that the E-SVR and GENIE3 algorithm predict directionality better than the E-EL algorithm. The plot also indicates that combining multiple ensemble feature importance algorithms is beneficial to the directionality prediction in the case of GENIE3+E-SVR (Wilcoxon rank sum test with continuity correction: GENIE3 and GENIE3+E-SVR, sample size 60, p-value = 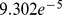).

### Computational Aspects

The ensemble subsampling approach used in NIMEFI results in modeling a regression problem(of sample size 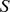 by 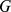 features), 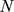 times, and using a feature selection algorithm of choice in each iteration to obtain a feature ranking. If an embedded feature selection technique is used, as was the case with the elastic net and support vector regression techniques, the cost breaks down to solving the 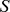 by 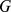 regression problem 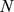 times, which is entirely dependent on the machine learning technique and implementation which is used. As such, if for example 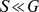, as is the case with gene expression compendia, using support vector regression in dual representation becomes computationally interesting. Furthermore, each iteration of the algorithm is independent and each target gene can also be solved in parallel. Due to these properties, running NIMEFI as a backfill job on a cluster becomes very interesting as the individual work packages can be tuned to almost every time slot.

In our work, we did not focus on optimizing the running time of the specific algorithms as we made use of general available libraries to compare a wide array of methods. However to give an indication of the relative running times, Table 7 gives an overview of the running times of some of the algorithms. These measurements were conducted using an Intel i3 CPU M350 clocked at 2.27 GHz, 8.00 GB of RAM memory and a 64-bit operating system. For the theoretical computational complexity, we refer to the specific software packages as listed in the Materials and Methods section.

**Table 7:** Comparison of indicative running times of E-SVR, E-EL and GENIE3.

Method	Running time DREAM4 (s/subsample)	Running time DREAM5 (s/subsample)
GENIE3	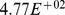	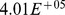
E-SVR	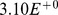	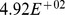
E-EL	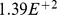	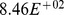

Run time in seconds for a single complete iteration or subsample of the algorithm considering all genes.

## Discussion

In this article we generalized the GENIE3 algorithm for GRN inference to other feature importance scoring algorithms in the same regression context. We presented a subsampling approach which allows any feature selection algorithm that can produce a ranking to be cast into an ensemble feature importance scoring algorithm. Using this scheme, we have analyzed the performance of several FS algorithms using the DREAM4 multifactorial and DREAM5 benchmarks, as well as several other artificially simulated datasets created using the SynTReN and GeneNetWeaver tools. We show that using this approach, several algorithms achieve equally good performance than GENIE3, demonstrating that an ensemble setting is key to achieve state-of-the-art-performance on the network inference task. In the DREAM5 challenge an ensemble of different network inference methods outperformed the single methods, establishing the ‘wisdom of the crowds-approach’ to the gene regulatory network inference problem. Motivated by these conclusions, we explored the method of using a rankwise combination of several ensemble feature importance scoring algorithms to the network inference task as opposed to a single one, an approach which we named NIMEFI.

On the DREAM4 dataset, the performance of the combined predictions outscores the single predictions, resulting in an overall score better than TIGRESS and the original GENIE3 algorithm. Using the artificially created datasets these finding were confirmed. Although no clear winner could be found among the different EFS algorithms, their combined predictions achieved a significantly higher AUROC score on all three datasets.

The results on the DREAM5 dataset were inconclusive. On the artificial dataset GENIE3 outperforms all other single algorithms but the NIMEFI combinations achieve a slightly better AUROC score at the cost of a slightly lower AUPR. On the biological dataset, all three individual methods perform similar and the NIMEFI combinations achieve a minimal performance gain.

Comparing the different methods further, we have shown examples in which the performance of the individual ensemble methods can be very similar although the predicted networks are different with respect to the node degree distribution or the predicted individual edges. Moreover, we have also investigated the ability of the methods to predict the correct directionality of the link.

We also explored the impact of the parameters of the proposed subsampling approach on the performance. We suggested and motivated default values and have shown that within reasonable and intuitive ranges, the performance is stable with regard to these settings.

Concluding, our findings indicate that the use of NIMEFI as opposed to using a single ensemble feature importance can further increase performance on the network inference task, reaching state-of-the-art performance on the DREAM datasets as well as on several artificial datasets. Combining the good performance with the computational attractive parallelization nature of NIMEFI, we believe our approach is an interesting alternative to other GRN methods. An implementation of our method is available for download at http://bioinformatics.intec.ugent.be/.
